# Mitochondria as a Disease-Relevant Organelle in Rheumatoid Arthritis: A Key Breakout in Fight Against the Disease

**DOI:** 10.3390/biomedicines13071708

**Published:** 2025-07-13

**Authors:** Antonella Iaconis, Francesco Molinari, Roberta Fusco, Rosanna Di Paola

**Affiliations:** 1 Department of Chemical, Biological, Pharmaceutical and Environmental Sciences (ChiBioFarAm), University of Messina, Viale F. Stagno d’Alcontres 31, 98166 Messina, Italy; antonella.iaconis@unime.it (A.I.); rfusco@unime.it (R.F.); 2 Department of Veterinary Sciences, University of Messina, Viale SS Annunziata, 98168 Messina, Italy; francesco.molinari@studenti.unime.it

**Keywords:** chronic autoimmune response, rheumatoid arthritis, chronic synovial inflammation, mitochondrial dysfunction, DMARDs

## Abstract

Rheumatoid arthritis (RA) is one of the most representative autoimmune diseases. The peculiarity of this disease is synovial inflammation, which results in joint destruction and often disability. Although there are still several pathogenetic mechanisms to be clarified, lately, most studies have highlighted the involvement of mitochondria in the onset and progression of the disease. Mitochondrial functions are connected to many metabolic processes and the delivery of proinflammatory mediators. Mitochondria play a crucial role in the physiopathology of RA, contributing to chronic inflammation, cartilage and bone injury and chronic autoimmune response. Mitochondrial activity influences many aspects of the disease that will be discussed in terms of their correlation with the onset and persistence of RA, starting from mitochondrial dynamics up to bone homeostasis, passing through DAMPs and affecting immune cell functionality. Recent therapeutic approaches aim to improve mitochondrial function, reduce oxidative stress, modulate mitochondria-mediated inflammation and restore energy metabolism homeostasis.

## 1. Introduction

**Figure 1:**
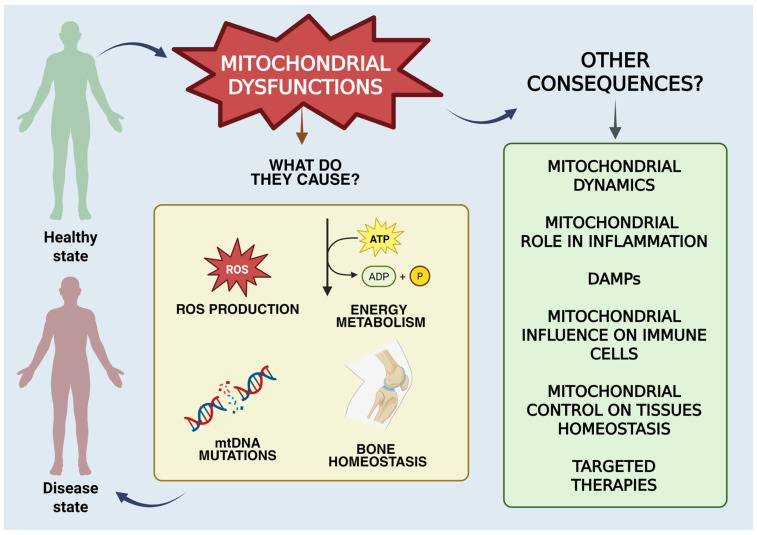
Graphical representation of the mitochondrial functions discussed in the manuscript. This figure was drawn using BioRender (https://BioRender.com, accessed on 9 July 2025), with the agreement license number GY28HNBEON. Illustration credit: Antonella Iaconis.

With reference to autoimmune diseases, rheumatoid arthritis (RA) is considered the most representative chronic inflammatory synovial disease, associated with the dysregulation of immune signalling pathways [1]. RA results in the stiffness and deformity of articular joints with inflammation of synovial tissues, neovascularization and autoantibody secretion, causing pain and difficulty in movement, increasing inconveniences to the patients’ health [2,3]. Currently, the aetiology is still unknown, and RA must be included among the possible factors that increase the risk of cardiovascular illness and connective tissue injuries, as well as compromising intestinal and oral health, causing many difficulties in the lifestyle and habits of affected patients [4]. The disease mainly affects people around 50 years old and affects women more than others, with a two-fold higher incidence [5]. RA is incurable nowadays and is often called “the cancer that never dies” for the peculiar characteristics of the disease like tissue destruction and erosion at the cartilage level, as well as the impairment of bone tissue. The disease also causes a percentage of disability in patients that is around 61.3%, which is quite high [6]. However, there are several approaches that can substantially reduce the pathological state and achieve drug-maintained remission [3]. Mitochondria act on several signalling pathways influencing immune cell activity, making this little organelle the heart of the activities that influence the emergence of diseases related to autoimmunity. A mitochondrial malfunction can result in mitochondrial DNA (mtDNA) mutations, abnormal increases in mitochondrial reactive oxygen species (ROS) and increased oxidative stress. Considering the numerous studies on the subject, it seems reasonable to affirm that mitochondrial malfunction or damage influence the pathogenesis and symptomatology of RA [1,7,8]. Its involvement goes beyond simple energy production, influencing several cellular processes, including immune response and inflammation processes [7]. This review will expose the main pathogenic pathways of RA. Then, we will focus on the theme underlying this work, which is understanding how mitochondrial functions act on the inflammatory processes related to RA, including a consideration of all mitochondrial dysfunction. Previous studies [9] highlight the main aspects of mitochondrial involvement in RA. However, this review provides an in-depth look at mitochondrial dynamics, DAMPs and mitochondrial metabolic activity that affects synovial cells, bone homeostasis and immunity. The aim is to provide a comprehensive view of mitochondrial involvement in RA (Figure 1). Finally, the main therapeutic pathways that aim to attenuate the symptoms and damages related to the dysfunction of mitochondria will be exposed.

## 2. Pathophysiology of RA

**Figure 2:**
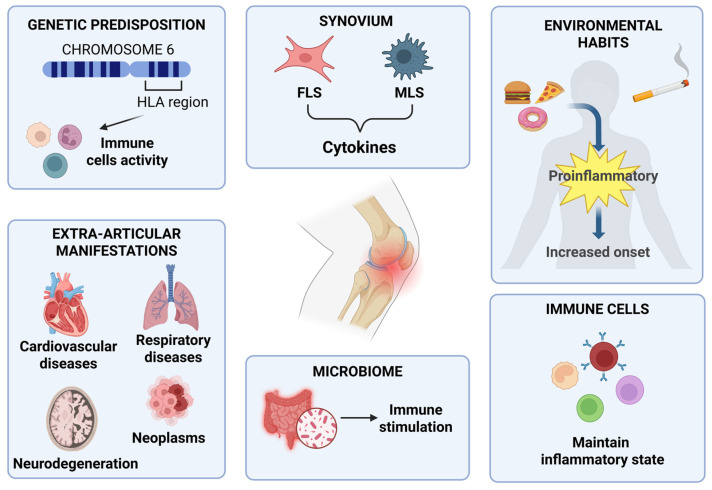
Graphic representation of the main aspects of RA pathophysiology [10,11]. This figure was drawn using BioRender (https://BioRender.com, accessed on 9 July 2025), with the agreement license number CB28HNBR66. Illustration credit: Antonella Iaconis.

The pathophysiology of RA is intricate and not yet fully understood but is believed to result from a combination of different factors that, separately or in conjunction, can influence the genesis and development of the disease [10,11]. RA is not a disease that comes on suddenly. The transition from immune health to autoimmunity involves a relentless inflammation of tissues and progressive remodelling of immune defence [10]. The main pathogenic features are joint erosion and joint-space reduction. The difficulty of treatment involves the failure of immune homeostasis as the inability to repair tissues once damaged [3,12]. RA is a heterogeneous disease, influenced not only by genetic factors but also by environmental factors and the microbiome (Figure 2).

### 2.1. Genetic Predisposition

The primary factor that influences the manifestation of the disease is assumed to be the existence of predisposition at a genetic level [12,13]. Many disease-associated genetic *loci* have been identified, over the order of hundreds, to be associated with predisposition to and subsequent onset of RA [2]. Between 70% and 80% of individuals affected by this type of autoimmune disease have particular types of antibodies called autoantibodies: specifically for RA, there are autoantibodies like rheumatoid factor (RF) and anti-citrullinated protein antibodies (ACPA). The RA risk *locus*, first identified around 1980, clarified the part of the HLA-DRB1 allele found within the human MHC on chromosome 6 [14]. The HLA region is linked to the immune response, as it contains class I and class II genes, the latter (HLA-DR, DQ and DP) producing the alpha and beta chains of the corresponding class II MHC molecule. This antigen-presenting molecule is accountable for the presentation of extracellular pathogens to T-cells [15]. Some disease-associated alleles have a conserved sequence of five amino acids (called the “shared epitope”), which lead to the erroneous presentation of autoantigens to lymphocytes by antigen-presenting cells (APCs), resulting in a T-cell-mediated autoimmune response [13,16].

### 2.2. Environmental Factors

Not only does the attendance of certain genetic *loci* cause the outset of the disease, but so does the environment, particularly lifestyle and eating habits [13]. Needless to say, as is well known from various points of view, cigarette-smoking has negative effects on health in general. Cigarette-smoking has negative influences on the regulation of adaptive and innate immunity [17]. Smoking patients have an increased risk of falling ill, with an estimated incidence between 20% and 30%, especially in RF-positive patients [18,19,20,21]. Cigarette smoke has a significant and negative impact on the disease, both by increasing the risk and worsening its course [22]. Immune response can be modulated by eating habits, as the diet can provide nutrients that can also interact with cells of immunity [23]. Generally, bad eating habits may facilitate arthritis onset due to the proinflammatory properties of some foods, but also due to metabolic disorders like insulin resistance, obesity or a combination of these factors [24].

### 2.3. Immune Trigger and Synovial Cells

Synovial tissue is often defined as tertiary lymphoid tissue (TLT). TLT has several analogies with secondary lymphoid tissue, where the maturation of lymphocytes T and B occurs, and which is connected to chronic inflammation [25]. Furthermore, due to pathogenetic changes of the synovial membrane in RA, there are two types of synoviocytes, such as macrophage-like synoviocytes (MLSs) and fibroblast-like synoviocytes (FLSs). These typologies of cells are important producers of molecules like cytokines. MLSs produce proinflammatory cytokines, including IL-1, IL-6 and tumour necrosis factor-α (TNFα). FLSs influence the synthesis of matrix metalloproteinases (MMPs) and other molecules involved in various inflammatory processes [26]. FLSs, along with antigen-presenting cells, are also bindweed in the making of *pannus* at cartilage–bone interfaces, mediating damages to and the erosion of bone and cartilage tissue [27].

### 2.4. Influence of the Microbiome

The immune system exists at the mucosal level because of a high load of microbial antigens, implying a causal relationship between microbiome impairment and RA pathogenesis [28,29,30]. A disturbed gut microbiota leads to the release of cytokines that promote the state of inflammation and cause a reduction in anti-inflammatory cytokines [31,32]. For example, microbial antigens can be presented to CD4+ T-cells by dendritic cells and macrophages, resulting in the differentiation of inflammatory T-cell subtypes and the production IL-17 [33]. It is important to clarify that it is not only T lymphocytes that participate in the outset of the pathology. B-type lymphocytes, or simply B-cells, can be stimulated by microbial antigens, contributing to the differentiation and production of autoantibodies characteristic of RA [31]. Thus, gut microbiota imbalance leads to inflammatory processes and an immune reaction that influences RA development [32,34].

### 2.5. Extra-Articular Manifestations of RA

RA is often associated exclusively with inflammation at the joint level. Extra-articular manifestations (EMs) and comorbidities are frequent in RA and involve cardiovascular and respiratory diseases, nervous system dysfunctions, gastrointestinal and renal disorders and even neoplasms [35]. RA patients show a high risk of developing cardiovascular disease (CVD) [36]. The causes of the high risk of myocardial infarction in RA are attributable to chronic inflammation, characteristic of the disease, which contributes to the development of atherosclerosis and other cardiovascular diseases such as congestive heart failure (CHF) [37,38]. In addition, CHF has been found to be more difficult to diagnose in patients with RA because they appear to have a more subtle initial symptom presentation. For example, myocarditis, related to CHF, is discovered in 11–50% of post-mortem examinations in patients with RA [35]. On the other hand, respiratory diseases are the second leading cause of mortality in RA patients, accounting for approximately 30–40% of them. It has even been found that pulmonary symptoms often represent the prelude to the onset of RA. In fact, chronic obstructive pulmonary disease (COPD) is a primary predictor of early mortality [39]. Regarding the nervous system, cervical myelopathy due to atlantoaxial subluxation is a common manifestation in RA patients [40]. However, neurodegenerative diseases such as Alzheimer’s disease and vascular dementia are also associated with chronic inflammation [41]. Furthermore, RA is associated with mood disorders such as anxiety and depression, with a prevalence of 21–70% and 13–20%, respectively [42]. Persistent inflammation and commonly used therapies cause gastrointestinal dysfunction. The most common are liver dysfunction, with elevated liver enzymes in 18–50% of RA patients. Amyloidosis, a complication of persistent inflammatory disorders, occurs in up to 13% of patients. Kidney disease, on the other hand, is most often related to complications of drug therapy [35]. Finally, it should be noted that RA patients have a slightly higher incidence of neoplasms than the general population, with an increased risk of Hodgkin lymphoma, non-Hodgkin lymphoma and lung cancer [43].

## 3. Overview of Mitochondrial Functions

**Figure 3:**
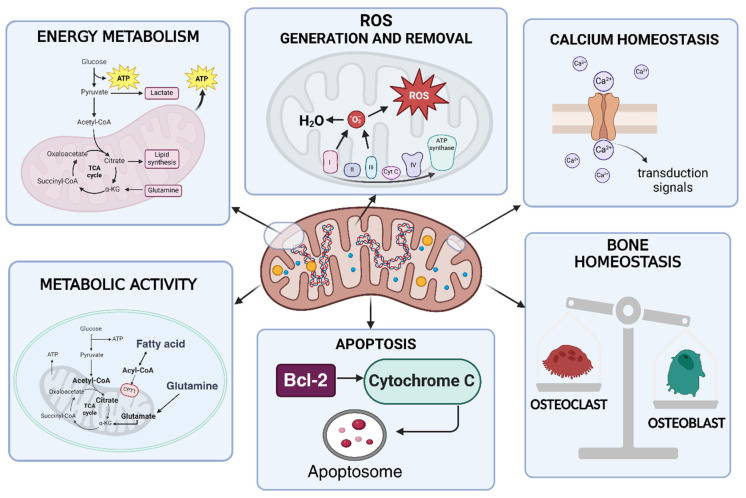
Mitochondrial functions. The mitochondrion, beyond being the centre of energy production, is responsible for the production and removal of ROS. It is also involved in calcium and bone homeostasis. The mitochondrion controls apoptotic processes. Fatty acid oxidation and glutaminolysis occur at the mitochondrial level [46]. This figure was drawn using BioRender (https://BioRender.com, accessed on 9 July 2025), with the agreement license number LI28HNC3GR. Illustration credit: Antonella Iaconis.

**Figure 4:**
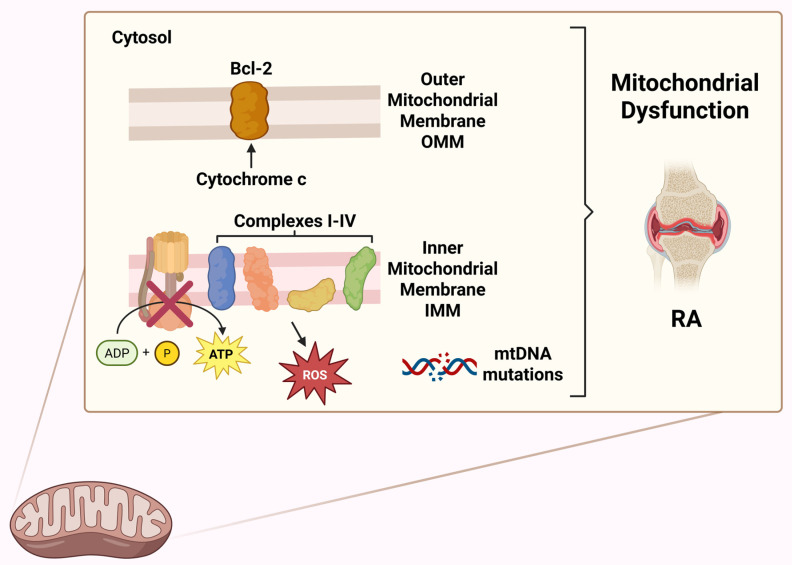
Graphical representation of the functions of mitochondrial compartments in RA [44,45]. This figure was drawn using BioRender (https://BioRender.com, accessed on 9 July 2025), with the agreement license number IN28HNCE5C. Illustration credit: Antonella Iaconis.

Emerging preclinical and clinical evidence, discussed in the following paragraphs, highlights the crucial role of mitochondria in the pathogenesis of RA. Mitochondrial function is involved in the development and pathogenesis of RA, as it represents one of the best-known autoimmune diseases. The different aspects of mitochondrial function will be discussed in the sections below. Mitochondria act as energy-producing plants of cells primarily by producing adenosine triphosphate (ATP) via the tricarboxylic acid cycle (TCA or Krebs cycle) and oxidative phosphorylation (OXPHOS). A double-membraned and semi-autonomous organelle, the mitochondrion has a unique structural organization. The structure is composed of a highly organized system of membranes, comprising an inner membrane, a membrane interstice and an outer membrane [44,45]. Mitochondria influence many other processes thanks to their ability to generate and, at the same time, remove the so-called ROS, as well as regulating cellular calcium levels and cell growth and facilitating programmed cell death at the metabolic level. Amino acid and lipid metabolism are also influenced by mitochondrial activities [46]. Figure 3 highlights the main activities and functions influenced by the mitochondrion. In Figure 4, some functions of the mitochondrial compartments in RA are schematically represented.

Mitochondria, often called the “cell’s power plant”, have a bacterial origin according to the endosymbiotic theory [47]. Their bacterial origins are easily seen in the fact that mitochondria have their own DNA (mtDNA), of a circular type and composed of 37 genes that play an essential role in maintaining their own functionality. The existence of this nucleic material enables us to classify the mitochondrion as a semi-autonomous organelle [48]. Along the ETC, the changeover of adenosine diphosphate (ADP) to ATP via inorganic phosphate occurs thanks to coenzymes that transport electrons, specifically nicotinamide adenine dinucleotide (NADH) and flavin adenine dinucleotide (FADH_2_), which produce water by allowing hydrogen atoms to detach as protons that combine with oxygen [49]. The ETC is formed by complexes I-IV and complex V, represented by ATP synthase. Cytochrome C and coenzyme Q are also part of OXPHOS [50,51,52]. Energy production is generally considered the main mitochondrial function. However, the ATP produced cannot be accumulated in any way but is constantly produced, also based on energy needs [53]. ATP production occurs in several stages, starting with glucose, which is split into pyruvate at the cytoplasmic level. Through oxidative decarboxylation, pyruvate (obtained from glycolysis) is converted into acetyl-CoA, which in turn becomes oxidized, producing CO_2_, FADH_2_ and NADH through TCA. By oxidative phosphorylation, the electrons transported through ETC complexes form a proton gradient, which allows ATP synthesis to produce ATP [54]. But the mitochondrion is responsible for numerous processes, which make it fundamental for the control of metabolism and cellular stress responses. During cellular respiration, mitochondria also produce ROS, which mainly act as cellular signals but, in high concentrations, can cause oxidative stress and cellular damage even at the mtDNA level [55]. Mitochondria, in physiological conditions, do not suffer numerous damages caused by ROS since they have specific systems that give them a clever DNA repair mechanism [56]. As anticipated, in apoptosis, or programmed cell death, mitochondria control cellular self-destruction, fundamental in maintaining the homeostasis of tissues [9]. Mitochondria degrade and recycle their components in response to damage that compromises their functionality. These processes are called mitophagy, which allow the autophagic degradation and recycling of mitochondrial components [57]. The mitochondrion is involved in cell communication, such as inducing cell death through cytochrome c release, ROS generation to activate transcription factors and the activation of immune cells due to mtDNA release. In addition, mitochondria control fission and fusion through 5′-adenosine monophosphate-activated protein kinase (AMPK) activation [9,58]. Calcium metabolism is modulated by mitochondria; they can store calcium ions, which are important in cell signalling and muscle contraction [59]. Mitochondria are also biosynthetic hubs of several important molecules, like amino acids, fatty acids and heme (a component of haemoglobin) [60,61]. Inflammasome is a protein complex bindweed in inflammation processes, and its activation is influenced by mitochondria through different mechanisms, like ROS release [9,48]. Mitochondria are implicated in autoimmune diseases because of their involvement in energy metabolism, which changes during the disease. This type of adaptation cannot happen without mitochondria, which can provide the energy needed to produce cytokines and antibodies by lymphocytes, for example. Macrophages or dendritic cells also require bioenergy for their activities [9,48]. It remains unequivocal that the mechanisms causing mitochondrial dysfunction are complex and involve numerous pathways, which is why it is appropriate to conduct more in-depth studies to develop new therapeutic approaches for diseases [9].

## 4. Mitochondrion Dynamics in RA

### 4.1. Fusion and Fission Dynamics

Mitochondrial homeostasis also regulates cellular activities. Beyond the innumerable functions that the mitochondrion regulates and influences, its functions undergo alterations if the structure of the mitochondrion itself is altered. In light of this, fusion (in which two or more mitochondria join to a larger, interconnected mitochondrion) and fission (as opposed to fusion, in which a mitochondrion splits into smaller mitochondria) play an indispensable function in keeping mitochondrial homeostasis, influencing all related processes [62]. Fusion and fission influence the effectiveness of immunological cells, and an incorrect balance between these two functions leads to a malfunction of the immunity response [57]. Studies conducted on human macrophages and on murine models, treated with lipopolysaccharide (LPS) or infected with mycobacterium tuberculosis, have demonstrated a fragmented mitochondrial state due to fission processes [62,63,64]. An increase in fusion processes influences human monocytes if treated with LPS [57,65]. Meanwhile, in lymphocytes, hyperfused mitochondria with narrow cristae have been found, which are capable of maintaining a high oxidative capacity and can produce ATP [66]. Multiple and interconnected factors are the basis of maintaining mitochondrial homeostasis. A study has highlighted the function of a particular type of microRNA; in fact, miR125b in monocytes regulates mitochondrial progress and apoptosis [57]. Although fusion/fission dynamics are widely studied, it has not yet been fully clarified what mechanisms of these processes contribute to increased RA pathophysiology [57]. Mitochondrial dynamics also influence stromal cells, particularly in synovial membrane fibroblasts. It is clear that a consequence of fibroblast proliferation is the formation of the so-called pannus, often defined as pathological tissue. Beyond this predominant consequence of fibroblast cell proliferation, this cell type is the cause of the degradation of the extracellular matrix of cartilage, and above all, they regulate and coordinate the recruitment and activation of the immune system. These events result in the worsening of pathological conditions. Mitochondrial activity determines the functional dynamics of FLS, making this cell type particular to the disease [67]. As previously mentioned, mitochondrial dynamics are modulated by multiple factors, which influence other cellular metabolisms and pathways, making it difficult to identify the specific causes of the disease [57].

### 4.2. Biogenesis and Mitophagy Processes

Mitochondria are dynamic organelles that undergo constant modelling through a series of interconnected processes that ensure their integrity and efficiency [68]. They are not only influenced by fusion or fission processes, but also by the new synthesis of mitochondria (biogenesis) and by the recycling and degradation of mitochondrial components through the so-called mitophagy process, which represents an autophagic process of mitochondrial components [57]. Therefore, mitochondrial homeostasis through fusion/fission or biogenesis/mitophagy is needed to maintain functional and active mitochondria and can represent indicative factors of cellular well-being. Metabolic signals like nutrient availability and mitochondrial membrane potential influence the processes that control mitochondrial homeostasis [69]. Furthermore, increased or decreased autophagic processes influence the pathogenesis of the disease [70,71,72]. In chondrocytes from osteoarthritis (OA) patients, the removal of defective mitochondria by mitophagy significantly improved cell survival, for example. It can also be assumed that in RA, mitophagy can prevent cartilage loss [73]. Compared to cells from healthy patients, mitochondrial mass is maintained constant despite the decrease in respiratory activity in T-cells derived from RA patients [74]. Indeed, the activation of the energy-sensing kinase AMPK is dysfunctional in this type of T-cell, suggesting that biogenesis and mitophagy are disrupted [75]. This may explain why mitochondrial mass in the T-cells of RA patients does not change [76].

## 5. Mitochondrial Metabolic Activity and ATP Production in RA

**Figure 5:**
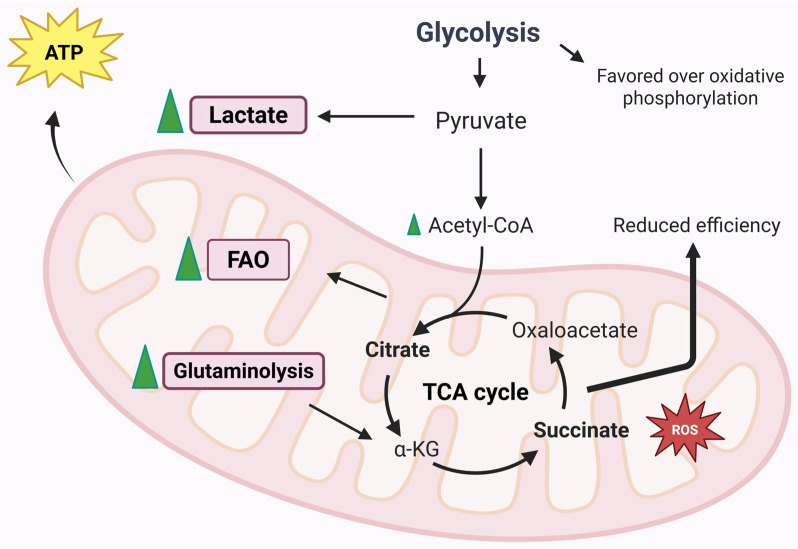
Mitochondrial energy regulation during RA [77]. This figure was drawn using BioRender (https://BioRender.com, accessed on 9 July 2025), with the agreement license number ZE28HNCP4V. Illustration credit: Antonella Iaconis.

Metabolic activity and ATP production play a surprisingly significant and increasingly recognized role in RA. The chronic inflammatory environment and altered metabolic demands of cells involved in pathogenesis influence the functioning of mitochondria [77]. As previously mentioned, the mitochondrion is the energy centre of the cells. Thanks to oxidative phosphorylation, carried out by the ETC, ATP is synthesized in large quantities. The enzyme ATP synthase produces ATP from ADP phosphorylation, thanks to the electrochemical gradient that is created across the internal mitochondrial membrane (mitochondrial membrane potential) [78]. It is therefore appropriate to specify that ATP production processes adapt to physiological needs [79]. Figure 5 represents mitochondrial energy regulation during RA.

### 5.1. Glycolysis

The first element to take into consideration is certainly glucose metabolism, which has recently been discovered to have a central role in pathogenesis [80]. In pathological conditions, and in particular in the inflammatory state that characterizes the articular joints in RA, the amount of lactate is greater than the amount of glucose, because the activity of glyceraldehyde 3-phosphate dehydrogenase (GAPDH) and lactate dehydrogenase (LDH), the main enzymes of the glycolytic pathway, is increased in synovial cells [81]. In fact, in the studies carried out by Garcia-Carbonell et al., it is clear that glycolysis is favoured over OXPHOS in FLS [82]. Another parameter to take into consideration is the amount of oxygen consumed during mitochondrial respiration, which can be evaluated to establish whether the activity of the respiratory chain is correct or not [78]. Small amounts of ATP are produced through glycolysis alone; in fact, its main purpose is to generate pyruvate or lactate but without consuming oxygen. Indeed, aerobic glycolysis is strongly linked to the inflammatory activity of immune and stromal cells [78,83].

### 5.2. Fatty Acid Oxidation

In addition to carbon sources from glycolysis, the TCA cycle can derive carbon from fatty acids through the generation of acetyl-CoA via the process of fatty acid β-oxidation (FAO), which occurs within the mitochondria and leads to the generation of NADH and FADH_2_, capable of driving the respiratory chain [57]. It is still unclear how fatty acid metabolism is disrupted during disease, which is why more in-depth studies are needed to understand FAO dysfunction in different cell types. In fatty acid metabolism dysfunction, a chemokine, CCL20, seems to be involved, which is responsible for lymphocyte recruitment and osteoclast bone resorption activity. A link between a dysfunction of fatty acid transport via carnitine and CCL20 production has been described. It has been shown that in cultures of human monocytes treated with exogenous carnitine, there is an increase in CCL20 production. The authors proposed that monocyte entry into the hypoxic and inflamed synovial joint alters fatty acid metabolism and leads to increased CCL20 production, resulting in a worsening of the inflammatory condition and increased joint damage [84]. Fatty acid metabolism is not modulated in the same way in the different cell types. In fact, different amounts of carnitine have been detected in synovial fluid samples from patients affected by the disease compared to healthy subjects. In addition, the enzymes involved in fatty acid β-oxidation, specifically Hydroxyacyl-CoA Dehydrogenase (HADHA) and acyl-CoA dehydrogenase (ACADVL), were downregulated compared to those in healthy subjects [85]. An increase in FAO enzyme expression was found following HIF-1a silencing in FLS, due to the hypoxia that characterizes the synovial tissue [85]. Disrupted fatty acid metabolism is evident in RA T-cells. Lipid droplet accumulations have been found in this cell type, due to a high rate of fatty acid synthesis with consequent T-cell hypermotility and tissue invasion. In addition, FAO enzymes are elevated in RA T-cells, but there are no significant differences in CPT1 expression [86]. It remains unclear how mitochondrial fatty acid distribution and degradation are related to FAO insufficiency and low ATP levels in RA T-cells [87]. Patients with the disease have a lower amount of fatty acids than patients with other types of autoimmune diseases, despite the fact that there are studies in the scientific literature that state that the quantities of free fatty acids are increased in patients affected by RA [88,89].

### 5.3. Glutaminolysis

Glutamine is an amino acid that donates nitrogen to produce nucleic acids and non-essential amino acids. This is not the only role of glutamine: it can also provide energy and precursors for biosynthesis thanks to a process known as glutaminolysis. The first step of glutaminolysis is catalysis by the mitochondrial enzyme glutaminase 1 (GLS1). GLS1 enzyme expression in RA-FLS patients is higher than in osteoarthritis patients. A lower amount of glutamine could potentially inhibit FLS proliferation. Even at the pharmacological level, the inhibition of GLS1 leads to a reduced proliferation of FLS, improving the symptoms of the disease [90]. Although these findings may influence the proliferative processes of FLS, they do not appear to be correlated with cytokines and metalloproteases [90,91]. For example, Th17 cells are the T-cell type that is mostly dependent on glutamine metabolism; in fact, GLS1 is positively regulated in this subset [57,92,93]. CD8+ are also influenced by glutamine metabolism. Indeed, CD8+, at low glucose levels, can metabolize glutamine into lactate. It is easy to understand that in inflammatory conditions, a competition for glucose is established, making the role of glutamine crucial [94]. Monocytes and macrophages are also influenced by glutamine. Macrophages treated with LPS and IL-4 showed an increase in the TCA cycle, thanks to glutamine-derived metabolites, α-ketoglutarate succinate and fumarate, which influence macrophage polarization and immune memory regulation [57]. However, more in-depth investigations into the mechanisms linking glutamine metabolism and RA pathogenesis are needed so that future therapies can also be developed.

### 5.4. TCA Cycle Metabolites

As is widely known, the mitochondrial matrix is the site of TCA. TCA is represented by a series of enzymatic reactions that aim to produce coenzymes with the aim of transporting high-energy electrons in the forms of NADH and FADH_2_, which are needed to feed the ETC. TCA-derived metabolites participate in micro- and macromolecule biosynthesis, which can have different functions, including signalling roles [78]. These metabolites can contribute to the onset and progression of the disease and can be used as targets for potential therapies. In addition, they can also be used as biomarkers that can provide information on the mechanisms of the disease.

#### 5.4.1. Succinate

Succinate is included in the metabolites produced by the TCA cycle and is bindweed in several mechanisms related to RA. It is not possible to consider succinate as a biomarker of RA, and studies performed on several patients with different degrees of inflammation highlight no differences between plasma and serum levels of succinate [95,96]. In murine models, the amount of extracellular succinate in the synovial fluid was elevated, especially in mice with paw swelling [97]. The mechanisms that regulate the quantity of succinate between blood circulation and the synovium are unclear, or differences in this metabolite are not detected in serum samples yet. The high amounts of succinate in the synovial environment are based on the different cell types. Macrophages accumulate succinate during inflammatory processes and release succinate into the extracellular environment following treatment with LPS [57,98]. Endothelial cells also accumulate succinate following exposure to LPS, but even in hypoxic conditions, an accumulation of the metabolite is also evident in FLS [99]. In CD4+ T-cells, the TCA cycle changes direction because of the impairment of the production of succinate [100]. This causes excess acetyl-CoA that is no longer converted to ATP, and CD4+ cells produce excess citrate that is transported out of the mitochondria into the cytosol. In addition, excess acetyl-CoA is involved in protein metabolism (post-translational modifications), impairing cell motility and cytokine production [100]. Being present in excessive amounts, succinate is present in the extracellular environment where it can be taken up by surrounding cells [101]. The GPR91 receptor, also known as SUCNR1 (Succinate Receptor 1), acts as a sensor for succinate and is expressed by monocytes/macrophages, granulocytes and also dendritic cells [97]. GPR91 is bindweed in the development of immune-mediated arthritis and stimulates the expansion of the Th17 cell population [101]. Acting as a signalling molecule, succinate can play a role in immunity, demonstrating that metabolic activity, mediated by mitochondria, is to be considered a determining factor in regulating the intensity of inflammation [102]. Macrophages accumulate succinate at intracellular levels, which results in the inhibition of prolyl hydroxylase (PHD) enzymes directly or through increased ROS generation [57]. In macrophages, the activation of SUCNR1 increases HIF-1α protein expression and IL-1β production. In antigen-induced arthritis models, the deletion of the Sucnr1 gene showed a reduced level of synovial IL-1β, demonstrating that SUCNR1 is involved in the inflammatory processes in RA [97]. Intracellular succinate accumulation is able to stabilize HIF-1α, as well as the activation of endothelial cell SUCNR1 by extracellular succinate. This allows the promotion of angiogenesis by promoting the production of vascular endothelial growth factor (VEGF) [99]. These events only favour the onset of the disease by promoting the recruitment and migration of leukocytes and promoting hyperplasia of the synovial *pannus* [103]. It should be noted that some studies have highlighted the anti-inflammatory effect of succinate [104]. It seems that the anti-inflammatory action is due to SUCNR1-dependent or independent mechanisms [104,105]. In fact, a study demonstrated that the macrophage expression of SUCNR1 is decreased by LPS treatment and increased by the cytokine IL-4 [105]. This result is in contrast with the findings of Littlewood-Evans et al. [97]. Currently, succinate-export mechanism from cells into the synovial environment are also unclear. Succinate is released into the extracellular environment following cell death, and this event is well-documented [57]. However, the release from living cells has not yet been documented and is therefore not fully understood. However, in tumour cells, succinate is taken up by sodium-coupled dicarboxylic acid transporters such as NaDC3 (SLC13A3) to fuel mitochondrial metabolism [106]. In active muscle cells, succinate can be carried by the monocarboxylate transporter MCT1 (SLC16A1) with important paracrine effects [107]. In both cases, transport is regulated by low pH levels [107,108]. It could be hypothesized that in the synovial environment, which has a low pH due to the high glycolic rate and the accumulation of lactic acid, the same transport mechanisms exist [109,110].

#### 5.4.2. Citrate and Aconitate

Citrate, like succinate, accumulates in several cell types [57]. Citrate is produced from acetyl-CoA and oxaloacetate by citrate synthase, a metabolite of TCA cycle. Citrate is also considered to be involved in the production of ROS since it is mainly used to produce NADPH (which, in fact, participates in the production of ROS) [111]. Citrate accumulates in inflammatory macrophages due to the downregulation of the downstream enzyme isocitrate dehydrogenase (IDH) [112]. This metabolite also accumulates in RA T-cells because of the deficiency of succinyl-CoA ligase and the inversion of the TCA cycle. Intracellular citrate can be converted to the immune-related metabolite itaconate by the enzyme aconitate decarboxylase 1 (ACOD1) [100,113]. Itaconate was originally known for its antimicrobial action, but a series of immunomodulatory functions have been highlighted, such as anti-inflammatory action through the inhibition of succinate dehydrogenase, or through the indirect activation of the transcription factor Nrf2 [114,115]. In synovial fluid samples, citrate levels were reduced compared to samples from healthy patients [116]. There appears to be a link between the decreased amount of citrate and the synthesis of the enzyme citrate synthase, as found in synovial tissue samples from RA patients. Indeed, other enzymes of the TCA cycle were also reduced in RA synovial tissue, including malate dehydrogenase and a component of the α-ketoglutarate dehydrogenase (DLST) complex. The reduction in these enzymes results in a compromise of the overall TCA cycle activity during the disease [85]. Itaconates influence both intra-mitochondrial functions (e.g., the inhibition of succinate dehydrogenase) and cytosolic functions (e.g., the alkylation of KEAP1 for activation of Nrf2). This metabolite is transported by the human citrate transporter and the 2-oxoglutarate/malate transporter through the mitochondrial membrane [115]. However, a definitive mechanism by which cells are able to excrete or take up itaconate has not been elucidated. In fact, itaconate is not able to freely cross membranes, so a study using various membrane-permeable modified forms of the metabolite was performed to determine its role in immune regulation [117]. It is still not clear how itaconate is absorbed from the extracellular environment and how this affects the disease [57]. For example, the inhibition of the enzyme succinate dehydrogenase could be attributed to the structural similarity of itaconate with succinate. It is therefore reasonable to state that itaconate could be considered a competitive inhibitor of the enzyme succinate dehydrogenase [114].

## 6. Mitochondrial Role in RA-Related Immune Inflammation

### 6.1. NOD-, LRR- and Pyrin Domain-Containing Protein 3 Inflammasome

The NOD-, LRR- and pyrin domain-containing protein 3, or simply the NLRP3 inflammasome, is an intracellular multiprotein complex that influences innate immunity and chronic inflammation and is considered an important factor for autoimmune disease onset [57]. This inflammasome is a complex formed by several proteins: NLRP3 (NOD-like receptor family, pyrin domain containing 3), which functions as a receptor to capture danger signals from pathogens, but also endogenous signals such as ROS and DAMPs; ASC (apoptosis-associated speck-like protein containing a CARD) protein, which connects NLRP3 to caspase-1; and caspase-1, which is a proteolytic enzyme that is activated by the inflammasome and which, in turn, activates the proinflammatory cytokines IL-1β and IL-18 [118,119]. Mitochondrial disruption leads to NLRP3 activation via several mechanisms. One of the main mediators of NLRP3 activation is ROS. ETC inhibition and the subsequent increase in ROS production by mitochondria led to the production of IL-1β, which indicates NLRP3 activation [120,121,122]. Not only do ROS influence NLRP3 activation; fatty acid metabolism, in which the mitochondrion is involved, is linked to the regulation of NLRP3 activation. NLRP3 and IL-1β expression is promoted by the mitochondrial uncoupling protein UCP2 obtained through a rise in the expression of the enzyme fatty acid synthase [123]. Several mitochondrial components promote NLRP3 inflammasome activation. The mitochondria-specific phospholipid cardiolipin is directly associated with both NLRP3 and caspase-1 and is critical for inflammasome activation, even to react to external stimuli such as pathogens [124].

### 6.2. Toll-like Receptor 9/Nuclear Factor Kappa B

Toll-like receptor 9 (TLR9) is a receptor that plays a role in the response of innate immunity. It is intracellular and carries out its protective function through several PRRs [125]. As already mentioned, dysfunctional mitochondria release their components that act as DAMPs and that can induce inflammatory processes through PRRs [126]. The overproduction of ROS and DAMPS by damaged mitochondria induces PRR activation and, consequently, the activation of TLR9, which trigger the inflammatory processes of the disease [127]. In fact, high levels of TLR9 are present in the serum of patients with preclinical RA [128]. In models of CIA, bionic nanogels depressed the TLR9 pathway, alleviating arthritis symptoms [129]. High levels of cell-free DNA (cfDNA) also activate TLR9 in the endosomes of immune cells, highlighting that the latter is also involved in pathogenesis. In this regard, methotrexate-loaded nanoparticle DNA scavengers (cNP-pp-PEG) have been used to improve the therapeutic effect of the drug by reducing its toxic effects [130]. TRLR9 can induce the activation of nuclear factor kappa B (NF-κB), which is a major factor of inflammation [131]. Activated NF-κB and HIF-1α induce a signalling cascade leading to ROS formation and M1 macrophage polarization [132]. In fact, a recent study has shown that the downregulation of the essential modulator of NF-κB (NEMO) modulates joint inflammation in CIA mouse models by inhibiting the NF-κB pathway [133]. This demonstrates that this factor also influences the pathogenesis of the disease, although further investigation is still necessary.

### 6.3. Cyclic GMP-AMP Synthase

Activated cyclic GMP-AMP synthase (cGAS), as a sensor of cytosolic double-stranded DNA (dsDNA), is an enzyme that catalyses the conversion of ATP and guanosine triphosphate (GTP) into cyclic guanosine monophosphate–adenosine monophosphate (cGAMP-a cyclic dinucleotide). cGAS is involved in the inflammatory process because it can stimulate the stimulatory adaptor protein of interferon gene (STING) and downstream pro-inflammatory factors on the endoplasmic membrane [134,135]. A study has highlighted that the accumulation of cytoplasmic dsDNA may represent a crucial cause for the disease, as it drives inflammation mainly via the cGAS/STING pathway in RA-FLS [136]. Oxidative damage, caused by an accumulation of ROS, also involves the release of mtDNA into the cytoplasm, which causes further activation of the DNA sensor cGAS-STING, facilitating downstream inflammatory processes [137,138]. cGAS-STING is able to activate NF-κB and NLRP3, increasing the production of multiple inflammatory factors, further worsening the inflammatory state [139,140]. TNFα also plays a crucial role in the development of RA, since it is able to inhibit mitophagy mediated by phosphatase and tensin homolog-induced kinase 1 (PINK1), causing mitochondrial damage. These events do nothing but increase the levels of cytoplasmic mtDNA and activate cGAS-STING [141]. Given its involvement in inflammatory processes, cGAS-STING represents a potential target for the development of therapies against RA.

## 7. Mitochondrial DAMPS

**Figure 6:**
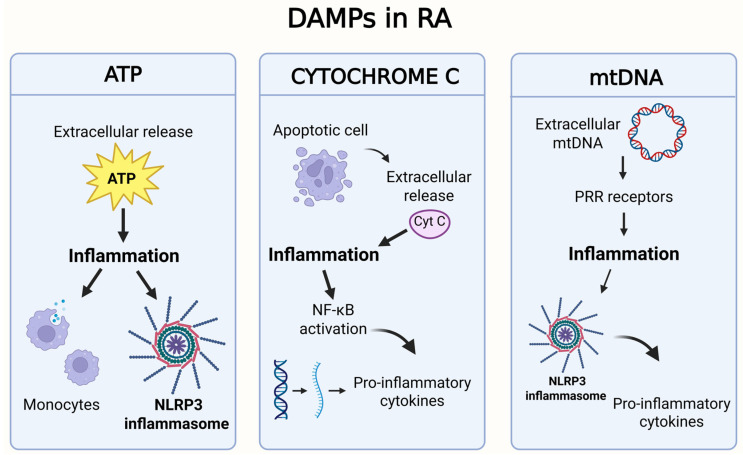
Graphical representation of the main DAMPs involved in RA [146]. This figure was drawn using BioRender (https://BioRender.com, accessed on 9 July 2025), with the agreement license number GD28HND677. Illustration credit: Antonella Iaconis.

Mitochondria are considered sources of so-called damage-associated molecular patterns (DAMPs). These endogenous molecules serve as signalling pathways for various signals, including tissue injury, cell destruction or cellular stress, and can activate immune cells in a manner similar to microbial pathogen-associated molecular patterns (PAMPs). Mitochondrial components can activate the same pattern-recognition receptors (PRRs) as exogenous PAMPs. Furthermore, DAMPs can signal through several receptors, including Toll-like receptors (TLRs), NOD-like receptors (NLRs), RIG-I-like receptors (RLRs) and purinergic receptors [142]. DAMPs can be released into the cytoplasm when mitochondria are damaged [7] and can bind to their corresponding receptors to elicit an innate immune response (Table 1). There are various types of DAMPs, including high-mobility histone B1, IL-1α and heat shock protein. The inflammatory response by the cells of the innate immune system is not only triggered by the release of DAMPs but also by the presence and release of mtDNA in the extracellular environment, leading to a whole series of consequences that are typical of the physiopathology of the disease [143]. Figure 6 shows the main DAMPs involved in RA.

**Table 1:** Schematic of the main DAMPs involved in RA.

DAMPs	Origin	Role in RA
ATP	Released from damaged or necrotic cells, or actively from stressed cells.	Extracellular ATP activates purinergic P2X receptors (particularly P2X7) on immune cells, leading to activation of the NLRP3 inflammasome and the release of IL-1β and IL-18. It contributes to synoviocyte proliferation and perpetuation of inflammation [144].
mtDNA	Circular DNA released from damaged or apoptotic mitochondria.	When released into the cytosol or extracellular space, it acts as a DAMP by activating PRRs. It promotes caspase-1 activation, IL-1β and IL-18 release, and contributes to the inflammatory positive feedback loop with mtDNA mutations [145].
Cytochrome c	In the cytosol, it is a key signal for apoptosis, but if released into extracellular space, it becomes a danger signal for the immune system.	When released into extracellular space, it is recognized by activating PRRs receptors. The interaction between cytochrome c and PRRs can trigger a cascade of pro-inflammatory signalling events, leading to the activation of pathways such as NF-κB and the release of pro-inflammatory cytokines (such as IL-1β, TNF-α, IL-6) [146].
HSP (heat shock proteins)	Released from damaged or necrotic cells.	Some members of HSP can act as DAMPs when extracellular, inducing inflammatory responses and stimulating cytokine production [147].

### 7.1. Adenosine Triphosphate

The mitochondrion produces 18 times more ATP than glycolysis alone [57]. The extracellular release of ATP, following cell death, stimulates immune cells by acting on several cell surface receptors [148]. ATP, which is released due to programmed cell death into the extracellular environment, has a chemo-attractive action on monocytes, functioning to facilitate the disposal of apoptotic debris [149]. Extracellular ATP release is connected to inflammatory phenomena because of the activation of the signals for the NLRP3 inflammasome [150]. In vitro, ATP is used as a signal for NLRP3 activation and the cleavage of pro-IL-1β and pro-IL-18 [118]. ATP activates NLRP3 through the coverage of the alternative purinergic receptor P2X7, which is a ligand-gated ion channel that participates in the regulation of the innate and adaptive immune systems [151]. P2X7 receptor activation by ATP results in potassium ion efflux, which serves as a mechanism for NLRP3 activation [118]. P2X7 receptor expression is elevated in RA patients compared to controls [152].

### 7.2. Cytochrome c

Cytochrome c plays a major role in apoptosis but is also known to function as a pro-inflammatory molecule when released into the extracellular space [153]. If cytochrome c reaches the extracellular environment, it can act as a DAMP and activate an immune response [142,154]. In murine models, an intra-articular injection of cytochrome c causes short-lived inflammatory arthritis in the animal [57]. Instead, cytochrome c in the serum of RA patients is lower than in healthy subjects. Furthermore, the levels of cytochrome c found in synovial samples from the same RA patients are also lower than the levels found in serum. This phenomenon could be justified by the consumption of cytochrome c in the inflammatory synovial environment being increased, although the role of this protein in the disease remains to be clarified [154]. Extracellular cytochrome c can also activate NF-κB, which regulates the gene expression of many pro-inflammatory cytokines. This activation contributes to the maintenance of the inflammatory process in RA [155].

### 7.3. mtDNA and Mutations

The proinflammatory properties of mtDNA have been demonstrated by Collins et al., who, through an intra-articular injection of mtDNA, induced articular inflammation in mice, while the injection of nuclear DNA produced no effect [57]. Extracellular mtDNA has been detected in both the synovial fluid and blood plasma of patients affected by the disease [156]. Biniecka et al. have suggested that hypoxia promotes mtDNA mutations [157]. Low oxygen levels result in oxidative damage to mtDNA and contribute to PRR activation [158]. The activation of NLRP3 and AIM2 inflammasomes is linked to the presence of cytosolic mtDNA, resulting in caspase-1 activation and the release of IL-1β and IL-18. Interacting with NLRP3, mtDNA can lead to the induction of apoptosis [159]. Higher levels of mtDNA mutations are present in synovial tissue from RA patients if compared to OA patients and/or healthy individuals, and many of these mutations involved amino acid changes [57,160]. Synovial levels of TNFα or IFNγ positively influence the frequency of mtDNA mutations [57]. Harty et al. have shown that inflammation promotes mtDNA mutations [160]. For example, in vitro treatment of RA-FLS with TNFα has shown an increase in the frequency of mtDNA mutation in an inflammatory environment, which, in turn, promotes mtDNA mutation. This creates positive feedback, which allows an increase in the inflammatory state by creating a sort of loop that persists in RA patients [57]. The number of mtDNA mutations in synoviocytes in patients with RA is approximately double that in patients with osteoarthritis [161]. Mitochondrial gene mutations are increasingly recognized for their potential role in the origin and propagation of RA. A summary of specific mitochondrial gene mutations implicated in RA are presented in Table 2.

## 8. Mitochondrial Role on Oxygen Availability in RA

### 8.1. Oxidative Stress

Synovial tissue undergoes an increase in cellular proliferation and, consequently, leads to a build-up of mitochondrial electron transfer. This results in a reduction in the availability of oxygen molecules to generate ROS, creating a hypoxic condition. In this particular type of condition found in synovial tissue, mitochondria undergo a change in functionality, structure and number, in response to oxidative stress and abnormal energetic changes [46,165,166,167]. The TCA cycle can be considered a metabolic hub and redox balance, and it produces metabolic intermediates that can be transported out of the mitochondrial membranes and participate in biosynthesis processes [168]. ROS are generated thanks to the loss of protons by ETC. During the transfer, the electrons in ETC will stay in unstable positions and are susceptible to oxidation by adjacent oxygen [169]. Mitochondria can be considered antioxidant defence systems and can maintain low levels of ROS, both those produced during cellular respiration and those produced by other cellular sources (e.g., macrophages) [46]. ROS production by mitochondria does not necessarily represent a pathological condition. In fact, approximately 2% of oxygen molecules can be used as a source to produce ROS. In chondrocytes, cells that constitute the cellular component of cartilage tissue, ROS are an essential component for cell physiology. Actually, ROS are necessary for chondrocyte repair and apoptosis, cytokine production and extracellular matrix synthesis [7]. Nevertheless, many chronic diseases are linked to oxidative stress due to an overproduction of ROS. In particular, the mitochondrial respiratory chain is responsible for the production of free radicals such as superoxide anion, hydroxyl radicals and hydrogen peroxide, which have strong oxidative power and are able to easily capture electrons from other substances harming DNA, proteins (and therefore enzymes) and lipids [165,170]. As anticipated, the organism has antioxidant systems of different nature, such as superoxide dismutase, catalase (CAT) and glutathione (GSH) peroxidase (GPX). In pathological conditions, the overproduction of ROS or a decrease in antioxidant systems is evident, leading to the establishment of oxidative stress with all its consequences [171]. In RA, ROS production is directly or indirectly involved in the induction of the involvement of other cells in the pathogenesis of the disease [166]. The hypoxic environment created at the synovial level favours the yield of free radicals that, in turn, induce damages to mitochondria and tissues [9]. For example, the presence of an excessive amount of ROS can promote the inhibition of the synthesis of important components of the extracellular matrix, thanks to the positive regulation and activation of metalloproteases. The consequence of this is evident with damage to the cartilage. Studies that have been carried out in vivo have in fact demonstrated that the activation of the nuclear receptor subfamily 1 group D member 1 (NR1D1) has lowered the expression of pro-inflammatory cytokines and matrix metalloproteinases (MMPs), leading to a reduction in synovial proliferation and a reduction in ROS production [172]. An important regulator of mitochondrial biogenesis is the peroxisome proliferator-activated receptor gamma coactivator-1 alpha (PGC-1α). During the inflammatory state, reduced levels of PGC-1α can modulate the expression of mitochondrial genes involved in antioxidant activity. This inhibition can promote the induction of oxidative stress and the activation of NF-κB [173]. PGC-1α activation is mediated by AMPK, which is able to phosphorylate specific enzymes that, in turn, have the ability to increase ATP production and decrease its consumption in low-energy conditions [76]. It is possible to say whether the AMPK–PGC1α axis plays an important role in joint inflammation [174]. Knowing the mechanisms by which ROS induce mitochondrial dysfunction may provide important input for the progression of specific targeted treatments.

### 8.2. Hypoxaemia

Oxygen availability affects mitochondrial function. Lack of oxygen, or hypoxia, leads to mitochondrial alterations at the structural and functional level, also affecting mtDNA and its stability [175]. The resulting hypoxia causes an increase in oxidative stress, mitochondrial damages eliciting further production of ROS, worsening the pathological state of RA [9]. In addition, in hypoxic conditions in human synovial cells, an increase in mitochondrial membrane potential (ΔΨm) is highlighted, which causes a metabolic shift to aerobic glycolysis and leads to an increase in the level of mutations affecting the mitochondria [176,177]. Moreover, hypoxia involves not only functional but also structural changes of the mitochondria. The hypoxic state involves the accumulation of mutations in the mtDNA, therefore leading to a state of total mitochondrial dysfunction [177]. Under hypoxic conditions, cells possess mechanisms that allow them to adapt to this environment. The so-called transcription factor hypoxia-inducible factor (HIF) is the main regulator of the response to hypoxia. The pathogenesis of RA is characterized by high levels of HIF-1α and HIF-2α. These factors have different roles: HIF-1α protects and maintains the dynamic equilibrium of the articular cartilage matrix, while HIF-2α is liable for matrix decomposition [9]. Again, factors involved in hypoxia may be targets of new therapeutic approaches.

## 9. Mitochondrial Influence on Immune Cells in RA

**Figure 7:**
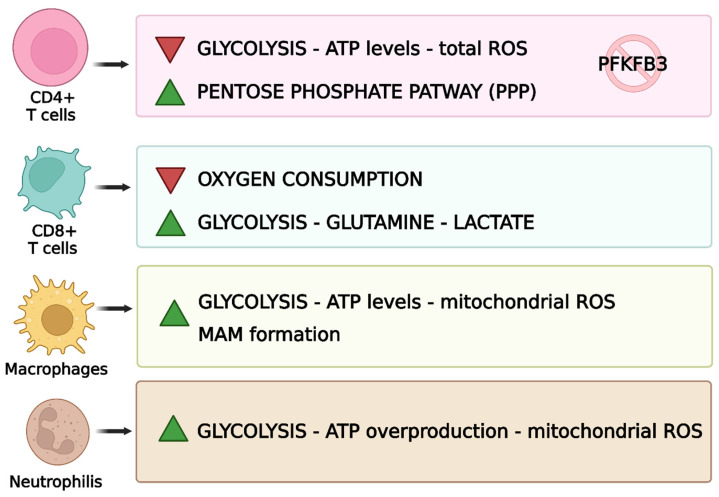
Mitochondrial functions related to immune cells [143]. This figure was drawn using BioRender (https://BioRender.com, accessed on 9 July 2025), with the agreement license number RO28HNDEFV. Illustration credit: Antonella Iaconis.

Mitochondria are involved in mechanisms that regulate the immune system (Figure 7) [143].

### 9.1. T-Cells

Mitochondria influence and regulate the activity of lymphocytes. Under physiological conditions, T-cells transform into effector T-cells and become proliferative, following antigen presentation by antigen-presenting cells (APCs) [7]. CD4+ T-cells are the main immune cells involved in RA [113].

#### 9.1.1. Metabolic Abnormalities in T-Cells

Immune system cells show metabolic abnormalities; in fact, CD4+ T-cells show mitochondrial insufficiency in RA, and low ATP generation and reduced ROS release are evident [178]. Regarding the glycolytic pathway, 6-phosphofuran-2-kinase/fructose-2,6-bisphosphatase 3 (PFKFB3), an enzyme responsible for the production of fructose 2,6-bisphosphate, which contributes to glycolysis, is inhibited during the disease [179]. On the other hand, glucose-6-phosphate dehydrogenase (G6PD), which can catalyse the pentose phosphate pathway (PPP), is upregulated. This phenomenon leads to the excessive production of NADPH and glutathione. These changes cause a reduction in the glycolytic processes and, consequently, ATP production [180]. Reduced amounts of ATP, due to its poor production, negatively affect the TCA cycle. This brings an excess of metabolites such as α-ketoglutarate, citric acid and acetyl-CoA, which affects the metabolism of T-cells and leads to an increase in the motility of this cell type, which leads to an increase in inflammation [181]. In addition, the excessive production of an antioxidant such as glutathione prevents the activation of cell cycle checkpoint kinase ataxia telangiectasia-mutated (ATM) and therefore prevents T-cells from regulating proliferation, favouring inflammatory processes due to excessive and uncontrolled proliferation of Th1 and Th17, worsening the pathological state [182]. In addition, AMPK, one of the proteins responsible for energy metabolism, is activated in physiological conditions when there is a drop in ATP, triggering mitochondrial biogenesis [75]. In RA T-cells, however, this mechanism does not work, and the mitochondria, already compromised, continue to function anyway [180]. CD8+ T-cells also play a role in RA; in fact, in a hypoxic environment and with low glucose concentrations, they increase glutamine uptake, leading to an increase in lactate production [94].

#### 9.1.2. Proinflammatory Cytokines in T-Cells

CD4+ is responsible for the production of interleukin IL-6 and therefore plays a relevant role in the onset of the disease, as it regulates the production of lymphocytes and consequently the inflammatory state [27,183]. Th17 lymphocytes also play a fundamental role in immunological modulation in RA, which are generally divided into a “pathogenic” class and a “non-pathogenic” class. Pathogenic Th17 regulates the positive immune response since they contribute to the production of proinflammatory cytokines, which specifically are IL-22, IL-17A and IL-17F. Non-pathogenic Th17 can secrete immunosuppressive factors such as IL-10 for negative immune modulation [184]. Furthermore, IL-17 release, in turn, leads to the production of proinflammatory cytokines such as TNFα, IL-1β and IL-6 in cartilage, synovial cells, macrophages and bone [185]. Despite numerous immunological studies conducted on T-cells, there are still several unanswered questions, which require greater attention, in order to develop targeted therapies in the future [27].

#### 9.1.3. Regulatory T-Cells

Regulatory T-cells (Treg) are essential for maintaining immune homeostasis and preventing autoimmunity [186]. Their main role is to suppress, directly or indirectly, other immune cell populations. Both the numerical and functional impairment of Tregs have been found in RA, contributing to the persistence of the inflammatory state [187]. In RA patients, mitochondria present alterations in morphology and a reduced expression of ECT elements. These abnormalities lead to a reduction in ATP production and an increase in mitochondrial oxidative stress (mtROS) [188,189]. Tregs, while having a protective function, may themselves be vulnerable to excessive oxidative stress. Excess mtROS may induce apoptosis in Tregs, further reducing their number and effectiveness in controlling autoimmune inflammation [190]. The accumulation of mtROS can induce post-translational modifications of proteins and damage to mtDNA, negatively affecting the expression and function of genes essential for the correct functioning of Tregs, leading to a worsening and persistence of inflammation [188,189].

### 9.2. B-Cells

It is important to specify that it is not only T-cells that are involved in the pathogenesis of the disease. B-cells also play a crucial role in the onset of RA [25]. This lymphocyte typology assumes a multifactorial role, since they are not only able to produce and secrete inflammatory interleukins and present antigen, but they also have the capacity to produce so-called autoantibodies. Autoantibody production contributes significantly to the physiopathology of the disease. The best-known and -studied autoantibodies, which play a major role in the development of RA, are rheumatoid factor (RF), but also anti-carbamylated proteins, anti-citrullinated protein antibodies (ACPA), anti-PAD-4, anti-GPI and anti-cyclic citrullinated peptide (anti-CCP) antibodies [191,192]. B-cells also function as antigen-presenting cells. This means that B-cells present the antigen they have acquired to CD4+ helper T-cells. In people with RA, a significant increase in two types of CD4+ helper T-cells has been found, namely follicular helper T (Tfh) cells and peripheral helper T (Tph) cells. These two types of T helper cells are able to secrete CXCL13 and IL-21, which influence the differentiation of B-cells themselves and consequently in the production of autoantibodies [193]. Furthermore, B-cells secrete and release a variety of different proinflammatory and bone-destroying cytokines, including TNFα, IFN-γ, IL-6, IL-1β, IL-10 and IL-70. For example, TNFα influences the expression of a particular type of receptor, the receptor activator of nuclear factor kappa-β ligand (RANKL), by B-cells in the presence of IL-1β, thus promoting the formation of osteoclasts, which are responsible for bone resorption [194].

### 9.3. Macrophages

In inflamed synovial tissue, a massive infiltration of macrophages is observed. These synovial macrophages (SMs) are considered key effector cells in the joint destruction and maintenance of the inflammatory state [195]. In RA patients, an increase in glycolytic processes and OXPHOS is evident in their macrophages, due to the increase in the inflammatory state [7]. In this case, mitochondrial dysfunction leads to excessive ATP production and therefore excessive oxygen consumption. RA macrophages can inactivate glycogen synthase kinase 3b (GSK3b), which regulates cellular respiration [196]. The inactivation of GSK3b leads to an increase in the formation of mitochondria-associated membranes (MAMs), which have the function of promoting calcium transfer for the correct functioning of mitochondria. The increase in MAM facilitates calcium transfer and increases ATP production by mitochondria [197]. In RA, an imbalance between the presence of proinflammatory M1 macrophages and anti-inflammatory M2 macrophages is evident, causing inflammation that leads to joint damage [198]. Indeed, during the disease, macrophages tend to polarize towards a pro-inflammatory M1 phenotype, which is also able to produce inflammatory cytokines such as TNF-α, IL-1β and IL-6 [199]. Immunoinflammation in RA amplifies glycolysis and oxidative phosphorylation, allowing for the consumption of large amounts of oxygen and the production of more ATP, favouring the formation of MAMs [200,201].

### 9.4. Neutrophils

Neutrophils also undergo changes due to mitochondrial dysfunction. Their activity depends on the availability of energy and therefore on glycolytic processes. They promote inflammation as they represent the source of autoantibodies, which are responsible for the inflammatory state and tissue damage. Due to mitochondrial dysfunction, there is an overproduction of ATP that improves the activity of neutrophils promoting inflammatory responses [202]. At present, the effect of the entire mechanism involving neutrophils on the disease is not well understood; however, it remains evident that they are also involved in inflammation and have compromised mitochondrial functions [7]. The presence of neutrophils in the synovial joint is characteristic of RA. The hypoxic environment and the production of ROS brings the activation and production of neutrophils [203]. The protein arginine deaminase 4, an enzyme that converts arginine to citrulline, when activated is expressed in neutrophils and drives the formation of so-called neutrophil extracellular traps (NETs) [204]. The process of NET formation is defined as NETosis. This process is fundamentally activated by ROS originating from nicotinamide adenine dinucleotide phosphate oxidase, whose activation depends on an increase in cytoplasmic Ca^2+^ concentration and occasionally on the generation of ROS in mitochondria [205]. NETs have an essential role because after sleep, they activate DAMPs, inflammasomes, APCs and T-cells [206]. Increased osteoclast formation from monocytes was found due to carbamylation in NETs, forming carbamylated NETs (cNETs) [207]. NETs could be a target for treatment: Liu et al. observed that using bovine serum albumin nanoparticles loaded with tretinoin, neutrophils underwent apoptosis and NET release was inhibited [208].

### 9.5. Dendritic Cells

Dendritic cells are APCs and play a critical role in initiating and maintaining immune responses. In particular, research highlights that it is the subpopulations of myeloid DCs in the synovial tissue that contribute most to the maintenance of the inflammatory state [209]. In RA, mitochondrial dysfunction leads to the excessive production of ROS and reduced energy efficiency. The ability of DCs to maintain immune tolerance is negatively affected by the oxidative and metabolic stress that arises due to mitochondrial dysfunction. This leads to the activation and polarization of T lymphocytes towards pro-inflammatory profiles [189]. In addition, mitochondrial dysfunction can trigger the NLRP3 inflammasome in DCs, promoting the release of inflammatory cytokines, which subsequently further amplifies the autoimmune response and tissue damage that characterize RA [210].

## 10. Mitochondrial Role in Tissues Homeostasis in RA

### 10.1. Apoptosis

Apoptosis, also defined as programmed cell death, is an essential process for maintaining tissue homeostasis, preventing excessive cell proliferation. Mitochondria play a fundamental role in the apoptotic process; in fact, they promote the oligomerization of pro-apoptotic proteins BAX and BAK on the outer mitochondrial membrane. Through the regulation of transmembrane potential and bioenergy depletion, the mitochondrion releases pro-apoptotic proteins, including cytochrome c, from the intermembrane space into the cytosol. At this point, cytochrome c binds and activates apoptotic protease-activating factor 1 (Apaf-1) and proteasome 9, leading to the formation of a complex known as “apoptosome”. The apoptosome is responsible for the cleavage and activation of caspase-3, which results in apoptotic vesicle formation [189]. Mitochondrial dysfunction negatively affects apoptotic processes and, consequently, synovial cell metabolism, maintaining the abnormal growth of synoviocytes and immune cells. [1,211]. In RA patients, the proinflammatory mediator AOPP accumulates and triggers a reduction in ROS produced in mitochondria by increasing BAX and cytochrome c in the cytosol, a phenomenon that triggers a decrease in the anti-apoptotic protein BCL-2 [212]. The control of apoptosis and, consequently, of cell proliferation influences the onset of the disease by acting on the proliferation of synovial cells and, consequently, on the formation of the *pannus* [176]. BCL-2 expression in RA synoviocytes increases in the presence of proinflammatory mediators such as TNFα and IL-1β. This has been observed in lymphoid aggregates, suggesting that there is a mechanism of protection from cell death of lymphocytes [177]. Thus, both RA synoviocytes and immune cells continue to survive in the inflamed tissue, thus perpetuating the inflammatory state.

### 10.2. Bone Homeostasis

Bone destruction is a hallmark and progressive feature of RA. Mitochondrial dysfunction significantly and deleteriously impairs the so-called bone remodelling by altering the balance between osteoblastic and osteoclastic activity [213]. Bone homeostasis is mainly controlled by two cell types, osteoclasts and osteoblasts. Osteoclasts are responsible for delivering substances that dissolve inorganic bone components and degrade bone matrix proteins. Osteoclasts are, therefore, the cells responsible for so-called bone resorption [214]. During the disease, the activity of these cells is closely linked to mitochondrial health, demonstrating that mitochondrial dysfunctions are also involved in bone homeostasis [189]. Mitochondrion influences osteoclast activity in several ways. Osteoclast differentiation is suppressed if OXPHOS in mitochondria is inhibited [215]. Mitochondria influence osteoclastogenesis by influencing osteogenesis-related signalling pathways. Excessive ROS promotes osteoclast proliferation and differentiation through pathways such as RANK/RANKL signalling [216]. Mitochondria control calcium homeostasis. Yinbo Wang et al. [217] demonstrated that MCU (primary calcium transporter within mitochondria) promotes osteoclast differentiation. The action of mitochondrial dysfunction on these aspects negatively affects bone absorption, further worsening the pathogenesis of RA [189]. Osteoblasts are specialized cells of bone tissue responsible for the formation and regeneration of bones, as they are able to synthesize and deposit the extracellular matrix of bone [218]. Osteoblasts require a considerable amount of energy to be able to synthesize high levels of collagen and bone matrix proteins [189]. It is, therefore, reasonable to think that mitochondrial dysfunction negatively affects the synthesis of new bone tissue, since a malfunction in energy production (ATP) leads to an derangement in the energy balance, such as to disadvantage processes that require a lot of energy [219].

### 10.3. Fibroblast Synoviocites

Mitochondrial dysfunction in FLS can drive chronic inflammation and increase oxidative stress in the disease because it promotes the release of proinflammatory cytokines, as in the case of impaired balance between NAD+ and NADH, which leads to inflammasome activation [7]. Due to this imbalance, inflammasome activation causes the maturation and release of TNFα, IL-1β and IL-18. The release of these cytokines aggravates the inflammatory condition by activating immune system cells in the synovial tissue [57]. Another factor influencing mitochondrial activity in FLS is TNF-like ligand 1A (TL1A). A study demonstrated that the stimulation of this factor in RA-FLS cells compromises the expression of ETC-related genes, impairing the functionality of the mitochondrial membrane potential, promoting mitochondrial dysfunction, and thus aggravating the inflammatory state. In addition, TL1A promotes the expression IL-6 and IL-8, but also the activation of inflammatory pathways involving matrix metalloproteinases, signal transducer and activator of transcription 3 (STAT3) and NF-κB, which further aggravate chronic inflammation [220]. In addition to the function, the structure of the mitochondrion is also affected, leading to the release of its genetic material (mtDNA) into the cytosol. The release of mtDNA, which acts as pathogen-associated molecular pattern (PAMP), due to its bacterial origin, leads to the activation of damage-associated molecular patterns (DAMPs), which, thanks to receptors such as Toll-like receptor (TLR9) or NLRP3 inflammasome, lead to the activation of immune system cells with subsequent release of proinflammatory cytokines [221]. An excessive release of cytokines such as IL-17 in the synovial membrane of rheumatoid individuals causes an excessive release of chemokines, prostaglandins and MMPs by FLS, leading to a stimulation of osteoclastogenesis and, consequently, to cartilage and bone destruction [222,223,224]. A more recent study demonstrated that IL-17 contributes to mitochondrial dysfunction by increasing ROS and disrupting ATP production [70]. Hypoxia also affects mitochondrial activity. The activation of HIF-1α due to the strong presence of proinflammatory cytokines in the synovium contributes to the onset of inflammatory state of the joint. Hypoxia affects glycolysis by causing a decrease in the expression of glucose 6-phosphate isomerase (G6PI) in the synovial tissue of RA patients, leading to an imbalance of metabolites. Due to the lack of oxygen, the TCA cycle loses functionality and, consequently, accumulates metabolites such as lactate and succinate. The accumulation of these metabolic intermediates leads to an increase in vascular endothelial growth factor (VEGF) secretion, which leads to an increase in the production of new blood vessels, a phenomenon found in the pathogenesis of various autoimmune diseases [225]. Another element that interferes with mitochondrial function is Ca^2+^. In RA, an incorrect efflux of Ca^2+^ is observed, which causes mitochondrial dysfunction. FLS exhibits increased mitochondrial Ca^2+^ uptake, which may affect the migration and invasion of FLS. An excess of Ca^2+^ ions in mitochondria leads to the opening of so-called mitochondrial permeability transition pores (mPTP). These pores represent voltage-dependent channels of the inner mitochondrial membrane, activated by the positive regulation of Ca^2+^ in the mitochondrial matrix. The abnormal opening of these channels promotes the release of ROS and various metabolites in the matrix, favouring a decrease in the membrane potential and inhibiting the processes of oxidative phosphorylation, thus compromising mitochondrial functionality [226]. ROS accumulation leads to the activation of matrix metal-proteasis MMPs through the upregulation of transcription factors, including NF-κB, activator protein-1 (AP-1) and MAPK [227]. The overexpression of some MMPs, typical of the synovial lining in RA, like MMP-2 and MMP-9, contributes to FLS migration and synovial hyperplasia, cartilage destruction and bone erosion [228]. In FLS, there are RA-associated mutations that regulate mitochondrial apoptotic function, such as Bcl-2-associated X protein (Bax), B-cell lymphoma 2 (Bcl-2) and p53. These mutations lead to the dysregulation of apoptotic processes, with a decrease in pro-apoptotic proteins and an increase in anti-apoptotic proteins [225]. During the disease, mitochondrial function in FLS is compromised, and consequently, all mitochondrial-related processes are damaged, including apoptotic processes. This results in cell survival despite being under stress or damaged [229].

### 10.4. Chondrocyte Autophagy

Mitochondrial dysfunction also affects chondrocyte survival because of the loss of mitochondrial integrity and functionality [230]. Mitochondria-mediated autophagy is a major apoptotic pathway in chondrocytes. PINK1/Parkin is the most well-known autophagic pathway. The depolarization of the mitochondrial membrane potential, typical of damaged cells, causes an increase in ROS production and a reduction in ATP production, releasing pro-apoptotic cytokines [69,231]. Counteraction dysfunction thus results from a dysfunction in mitochondrial-mediated energy metabolism, for example [232]. TNFα and IL-1 influence the generation of mitochondrial membrane potential, reducing the activity of MRC complex I, and thus are involved in cartilage degradation [233]. Counteraction dysfunction thus results from a dysfunction in mitochondrial-mediated energy metabolism. For example, high Ca^2+^ concentrations in mitochondria lead to cell death, while low concentrations lead to the disruption of cellular energy metabolism. A dysfunction in calcium homeostasis involves a movement of Ca^2+^ from the endoplasmic reticulum into the mitochondria via the inositol 1,4,5-triphosphate receptor (IP3R). In this way, mitochondrial function is impaired and pro-apoptotic signals are activated in chondrocytes [234]. Oxidative stress induces apoptosis through the accumulation of advanced oxidative reactions products (AOPP), which are considered important markers for RA patients. The overproduction of ROS, resulting in mitochondrial dysfunction, causes the activation of the cysteine family, activating endogenous apoptotic pathways in chondrocytes [235]. Mitochondrial dysfunction favours the disease by disrupting repair activity against cartilage degradation and promotes the production of oxidative stress further causing chondrocyte apoptosis [7].

## 11. Mitochondrial Therapeutic Approaches

Mitochondrial dysfunction is not the sole and sufficient cause for the disease but plays a fundamental role in triggering, exacerbating or perpetuating the chronic inflammation characteristic of the disease [7]. Immune-mediated inflammatory diseases have become better understood, especially over the past two decades. The main existing therapies aim to reduce the effects of the disease and improve the quality of life of patients [236]. Therapies against RA aim to reduce the inflammatory state in the joints by reducing the progression of lesions and irreversible bone destruction. Currently, there are drugs in clinical trials that aim to restore mitochondrial function. In essence, mitochondria are involved in the process by which drugs are metabolized. Nowadays, therapies do not target the mitochondrion, but have mitochondria involved in their treatment mechanism. These currently existing therapies aim to reduce inflammation (anti-inflammatory action) and modulate the immune system response [7]. In Table 3, relevant clinical and pre-clinical studies are shown.

RA is treated with so-called disease-modifying anti-rheumatic drugs (DMARDs), and there are several therapies that aim to restore proper mitochondrial function. Since mitochondrial dysfunction is not the primary cause of the disease, all the therapeutic approaches currently implemented against the disease are used in combination with the commonly used DMARDs [242]. As usual, the drugs used have side effects (Table 4) that often worsen the health conditions of those affected. The main categories of drugs currently in use against RA are presented below.

### 11.1. Conventional Synthetic Anti-Rheumatic Drugs (csDMARDs)

In the early stages of the disease, conventional synthetic DMARDs (csDMARDs) are used, which act by suppressing the immune system and slowing the progression of the disease. This type of drug reduces inflammation but does not behave as a nonsteroidal anti-inflammatory drug, but rather modulates inflammatory processes, bringing improvements in joint damage and, consequently, pain. Like all drugs, csDMARDs can also cause side effects, which vary depending on the drug used [7].

#### 11.1.1. Methotrexate

Methotrexate (MTX) is one of the most commonly used csDMARDs in the treatment of RA. It has a similar structure to folic acid (a B vitamin essential for DNA synthesis and cell replication) and aims to compete with the folate-dependent enzyme. This type of similarity acts at the level of cellular proliferation, decreasing it, and also involves a decrease in the synthesis of pyrimidines and purines [249]. MTX can be considered an immunomodulator and acts according to different mechanisms, including the inhibition of the enzyme dihydrofolate reductase (DHFR) and increasing adenosine production. DHFR reduces dihydrofolic acid into tetrahydrofolic acid, which in turn affects the synthesis of purines and some amino acids. Therefore, the inhibition of this enzyme leads to a decrease in the synthesis of RNA and DNA, consequently acting on cellular proliferation, with particular reference to immune cells. The increase in adenosine production by the action of MTX instead allows the suppression of the activity of immune cells by acting as an anti-inflammatory [250]. MTX also functions to reduce oxidative stress thanks to the modulation of ROS production by influencing mitochondrial membrane potential and improving mitochondrial function [7]. The perpetuation of oxidative stress can have inhibitory effects on monocytes and cytotoxic T-cells and induce T-cell apoptosis, contributing to the anti-inflammatory effects of the drug [7]. MTX induces mitochondria- and caspase-dependent apoptosis in cultured synovial cells [251]. MTX is nephrotoxic and can also lead to liver injury; this is a reason why it is necessary to find alternative forms of therapy, since those currently available have severe side effects [243,244,245]. The side effects of MTX, which can vary from patient to patient, range from gastrointestinal to liver disorders [252].

#### 11.1.2. Leflunomide

Leflunomide (LEF) is also a csDMARD and has side effects like MTX. LEF inhibits the mitochondrial inner membrane protein dihydroorotate dehydrogenase (DHODH), which helps inhibit cell proliferation by reducing pyrimidine synthesis [253]. LEF also interrupts cellular inflammatory signalling by inhibiting tyrosine kinase activity. Mitochondria undergo the effects of LEF, particularly in the process of mitochondrial fusion, because the drug influences the expression of mitochondrial fusion elements 1 and 2, promoting mitochondrial elongation so as to improve resistance to stress and allow cells to resist death [253]. The active metabolite of leflunomide is teriflunomide, which can also inhibit the activity of complex III of the respiratory chain, and therefore interferes with OXPHOS and aerobic glycolysis in activated T-cells [254]. In addition, the inhibition of both DHODH and complex III results in enhanced ROS production, inducing apoptosis through oxidative stress and therefore decreasing cell proliferation [255]. The most impactful side effects are at the hepatic level. Research conducted on hepatocellular carcinoma cell cultures (HepG2) demonstrated the cytotoxic effect of LEF. The cytotoxic effect of the drug involves lactate dehydrogenase (LDH) release and ATP depletion and also acts at the mitochondrial membrane level by depolymerizing them [246].

#### 11.1.3. Sulfasalazine

Another csDMARD is sulfasalazine, which can be considered one of the most commonly used in combination with other csDMARDs. Sulfasalazine exerts an anti-inflammatory effect by inhibiting purine synthesis and causing increased adenosine release and binding to adenosine A2-type receptors on the surface of inflammatory cells. This drug may lead to apoptosis induction in T-cells by altering mitochondrial permeability. In this case, apoptosis is partially mediated by apoptosis-inducing factor (AIF) [256]. This drug allows the mitochondrial nuclear translocation of AIF and the accumulation of Bax mitochondria that influence the release of AIF to eliminate inflammatory cells [7]. Thanks to this effect, inflammatory cells are eliminated, and consequently, the cell cycle activation loop that results in the establishment of chronic inflammation is interrupted. The main side effect of the drug has been demonstrated on in vivo models and mostly affects the renal system. Damage to the renal tissue is influenced by mitochondrial destruction, which is caused by an increase in ROS and lipid peroxidation (LPO)—due to mitochondrial depolarization—GSH depletion and swelling [247]. Some botanical extracts are used for the treatment of the disease. For example, Tanshinone IIA, which is extracted from the root of *Salvia miltiorrhiza*, has shown effects on RA-FLS because it has anti-inflammatory and pro-apoptotic properties and can regulate the protein related to apoptosis, included the release of mitochondrial cytochrome c and also the expression of Bcl-2, Bax and Apaf-1 [257]. Alternatively, resveratrol (Res), a non-flavonoid polyphenolic organic compound, can inhibit the production of ROS by activating the Nrf2 pathway in such a way as to prevent cell proliferation and induce apoptosis in RA-FLS [258].

### 11.2. Targeted Synthetic DMARDs (tsDMARDs)

Furthermore, thanks to studies carried out on signal transduction pathways, synthetic targeted DMARDs (tsDMARDs) have been introduced and act on specific intracellular pathways that influence the inflammatory state [12,259,260]. Among these, JAK inhibitors are the most widely used tsDMARDs.

#### JAK/STAT Pathway Inhibitors

The so-called JAK–STAT (Janus kinase-signal transducer and activator of transcription) signalling pathway performs countless functions and regulates various processes, including immune response. The main components of this type of signalling are Janus kinases (JAKs) and signal transducers and transcription activators (STATs) [261]. JAKs are intracellular tyrosine kinase enzymes that transmit signals that affect inflammation, and they influence the signal transduction of several proinflammatory cytokines. So, JAK inhibitors act intracellularly, blocking these enzymes. Furthermore, JAKs phosphorylate and consequently activate STATs, which in turn move to the cell nucleus and regulate gene transcription. Through this type of inhibitor, the activity of the JAK enzymes is blocked, interrupting the signalling cascade and reducing the inflammatory state [7,261]. JAK inhibitors have common side effects like headaches and nausea. However, there are more serious side effects, even including an increase in the risk of serious infections. Tofacitinib is the best-known JAK inhibitor drug. It has been shown that this drug acts on RA-FLS, leading to an increase in ATP production. The increase in energy production in turn causes a greater presence of ROS; furthermore, the drug acts on the regulation of genes linked to glycolysis and influences the potential of mitochondrial membranes [248]. There are also specific STAT inhibitors that aim to block this signalling pathway decreasing the action of JAKs. For example, STAT3 activation is associated with hypoxia and promotes HIF-1α activity [262]. However, at present, this type of therapeutic approach is still in its experimental phase.

### 11.3. Biological DMARDs (bDMARDs)

Therapeutic strategies involve biological DMARDs (bDMARDs) such as TNFα inhibitors and IL-6 receptor inhibitors, but also selective modulators of T-cell co-stimulation and drugs that reduce the number of B-cells. Again, this type of drug is often used when drugs belonging to the csDMARD class are not very effective. Among bDMARDs, tocilizumab is the most commonly used drug for refractory RA and is an IL-6 receptor antagonist that reduces oxidative stress. Oxidative stress is improved in patients undergoing tocilizumab treatment, demonstrating that the drug counteracts ROS production, and in particular, those induced by cytokines [263]. Tocilizumab also has some peculiar side effects, including infections and neutropenia, but mainly at the injection site, presenting subcutaneous tissue disorders [264,265]. Meanwhile, adalimumab and infliximab are used as monoclonal antibodies against TNFα. Thanks to the action of these drugs (like inhibiting hypoxia-induced mitochondrial mutations and oxidative stress), several peculiar characteristics of synovial tissues inflammation are inhibited [266]. A gene expression study performed on the peripheral blood of RA patients suggested that the modulation of mitochondrial activity has therapeutic effects [267].

## 12. Materials and Methods

All the research was carried out across three major databases, i.e., PubMed, Google Scholar, Web of Science and Scopus, focusing on studies published between 2015 and 2025, excluding earlier research to ensure up-to-date insights. The search strategy utilized free-text keywords, including “rheumatoid arthritis”, “chronic synovial inflammation”, “mitochondrial dysfunction” and “DMARDs”. This process produced a final selection of articles for study references. In order to include the most recent information on the subject, publications from between 2020 and 2025 were mainly selected. Initially, articles based on the terms “rheumatoid arthritis” and “mitochondrial dysfunction “ were selected for the chosen time period (2020–2025). From these, many were excluded because they were not reviews. This process produced a final selection of about 268 articles for study references.

## 13. Conclusions and Future Prospective

Mitochondria influence numerous cellular functions and can modulate the immune response. A small but multifunctional organelle involved in energetic, apoptotic and inflammatory processes plays a primary role in RA, not only in pathogenesis but also in the perpetuation of the inflammatory state. Alterations in mitochondrial homeostasis contribute to increasing and, in some ways, are the main cause of tissue damage and the progression and worsening of the autoimmune state of the disease. The main problem related to RA is linked to the dysfunction of a cellular organelle that has the ability to influence countless functions, making it difficult to develop a specific therapy. Furthermore, the role mitochondria play in apoptosis impedes the natural mechanisms of tissue homeostasis promoting cell proliferation. New therapies are progressing that are aimed at improving mitochondrial dysfunctions, which are arousing growing interest, in particular for targeted and specific therapeutic approaches, which prevent patients from struggling with the painful symptoms and the innumerable systemic side effects that current drugs cause. Nevertheless, the pathophysiology is multifactorial, and a definitive solution has not been reached yet. However, developing therapies that specifically target the processes that promote the onset and mostly worsening of the pathology could be a good solution, considering that the therapies currently available have numerous side effects that, over time, could worsen patient health and lead to the development of further problems.

## Figures and Tables

**Table 2:** Mitochondrial gene mutations implicated in RA.

Mitochondrial Gene/Locus	Mutation Type/Description	Role in RA
mtDNA	Increased frequency of somatic mutations in mtDNA in synovial tissue.	Mitochondrial dysfunction, increased ROS and release of mtDAMPs, driving chronic synovial inflammation. Mutations also lead to impaired ATP production and altered cellular signalling [57].
MT-ND1 (NADH dehydrogenase 1)	Somatic mutations in this gene, encoding subunit one of Complex I of the electron transport chain.	Mutations in MT-ND1 can lead to dysfunctional Complex I, impairing oxidative phosphorylation and increasing ROS production [162].
Mitochondrial D-loop Region	Various point mutations and deletions.	The D-loop is a non-coding region involved in mtDNA replication and transcription. Mutations here can affect mtDNA copy number and overall mitochondrial function [163].
Genes encoding tRNA or rRNA	Point mutations in genes for mitochondrial transfer RNAs (tRNAs) or ribosomal RNAs (rRNAs).	Mutations in these genes can impair mitochondrial protein synthesis, leading to widespread defects in the electron transport chain complexes and other mitochondrial proteins [164].
Genes of the ETC	Mutations in genes encoding other subunits of Complexes I-IV and ATP synthase.	Dysfunction in any part of the ETC can lead to inefficient ATP production and increased electron leakage, resulting in higher ROS levels, worsening the inflammatory state [7].

**Table 3:** Relevant clinical and preclinical studies on mitochondrial dynamics and RA (with PICO application).

Patient (P)	Intervention (I)	Comparison (C)	Outcome (O)	Study Design	Trial Registration Number
RA patients	Drugs that directly modulate mitochondrial dynamics	Placebo or standard therapy	Reduction in disease severity, inhibition of inflammatory mediators. Restoration of mitochondrial morphology, reduction in migration and invasiveness. Mainly pre-clinical phase [57].	Preclinical studies (mouse models of CIA) and in vitro studies (RA-FLS) [57].	Currently unavailable
RA patients	JAK inhibitors	Placebo or conventional/biologic DMARDs	Improved patient-reported outcomes. Mitochondrial implications: some studies suggest that JAK inhibitors may modulate the energy metabolism of immune cells, including mitochondria. Status: numerous trials completed with positive results and approved drugs [237].	Preclinical studies [237].	NCT01359150 (Tofacitinib) NCT01721057 (Baricitinib) NCT02706852 (Upadacitinib)
RA patients	Statins (e.g., Atorvastatin)	Placebo or standard therapy	Statins may modulate mitochondrial function (e.g., reduction in ROS formation and decreased swollen joints). Status: research is focused on modulating mitochondrial dynamics as the primary endpoint [238,239].	In vitro and preclinical studies [238,239].	NCT06841536 (Statins) ISRCTN41829447 (Atorvastatin)
RA patients	Targeting the mitochondrial calcium uniporter (MCU)	Standard therapy	Inhibition of MCU reduced migration and invasiveness of RA-FLS, restored altered mitochondrial dynamics and reduced mitochondrial ROS. Status: primarily preclinical research not registered [240].	Preclinical studies (mouse and FLS models of RA [240].	Currently unavailable
RA patients	Matrine (traditional Chinese medicine compound)	Placebo or standard therapy	Evidence of efficacy in animal models and some initial clinical studies in RA. Status: research in progress [241].	Limited preclinical and clinical studies [241].	No specific registered trials were found

**Table 4:** Main side effects of drugs currently used to treat RA.

Category	Drug	Main Side Effects
Conventional Synthetic DMARDs	Methotrexate	Nephrotoxic [243,244,245] Hepatotoxic [243,244,245]
	Leflunomide	Hepatotoxic [246]
	Sulfasalazine	Nephrotoxic [247]
Targeted Synthetic DMARDs	Tofacitinib	Headaches [248] Nausea [248] Risk of infections [248]
Biological DMARDs	Tocilizumab	Subcutaneous tissue disorders [232] Neutropenia [233]

## Data Availability

Not applicable.
